# Expression of Stem Cell and Epithelial-Mesenchymal Transition Markers in Circulating Tumor Cells of Breast Cancer Patients

**DOI:** 10.1155/2014/415721

**Published:** 2014-05-08

**Authors:** Natalia Krawczyk, Franziska Meier-Stiegen, Malgorzata Banys, Hans Neubauer, Eugen Ruckhaeberle, Tanja Fehm

**Affiliations:** Department of Obstetrics and Gynecology, University of Duesseldorf, Moorenstraße 5, 40225 Duesseldorf, Germany

## Abstract

Evaluation and characterization of circulating tumor cells (CTCs) have become a major focus of translational cancer research. Presence of CTCs predicts worse clinical outcome in early and metastatic breast cancer. Whether all cells from the primary tumor have potential to disseminate and form subsequent metastasis remains unclear. As part of the metastatic cascade, tumor cells lose their cell-to-cell adhesion and undergo epithelial-mesenchymal transition (EMT) in order to enter blood circulation. During EMT epithelial antigens are downregulated; thus, such tumor cells might elude classical epithelial marker-based detection. Several researchers postulated that some CTCs express stem cell-like phenotype; this might lead to chemoresistance and enhanced metastatic potential of such cells. In the present review, we discuss current data on EMT and stem cell markers in CTCs of breast cancer and their clinical significance.

## 1. Introduction

Presence of disseminated tumor cells (DTCs) in bone marrow and circulating tumor cells (CTCs) in peripheral blood of primary breast cancer patients was shown to be associated with impaired clinical outcome [1, 2]. Moreover, the persistence of CTCs/DTCs after completion of adjuvant treatment also represents a negative prognostic factor [3–5]. These cells are therefore assumed to be a surrogate marker of minimal residual disease and precursors of distant metastasis. Despite the prognostic relevance of tumor cell dissemination, detection of tumor cells in blood or bone marrow is not necessarily followed by relapse of disease. While most of these cells are already apoptotic or dead and others will successfully be eliminated by shear forces of the bloodstream, only a small group of CTCs possesses the ability to extravasate and migrate through the endothelial cell layer [6–10]. Merely a fraction of those is able to survive at secondary sites and cause tumor growth “metastatic inefficiency” [11, 12]. Although factors determining the fate of CTCs still remain to be elucidated, one presently discussed theory considers epithelial-mesenchymal transition (EMT) to be a crucial step in tumor cell dissemination.

EMT is a phenomenon hypothesized to contribute to cancer progression and metastasis [13]. In this process epithelial cells of the primary tumor undergo a series of phenotypic changes, such as reduction of cell-cell adhesion, increment in cell mobility and invasiveness, loss of epithelial markers, and acquisition of mesenchymal phenotype [14]. Moreover, it has been demonstrated that the process of EMT can generate cells with stem cell-like properties [15]. Cancer cells with stem cell-like, self-renewal capabilities (cancer stem cells: CSCs) are currently regarded to be the source of metastatic tumor spread [16]. Since CTCs have been shown to express mesenchymal and stem cell markers, it has been recently postulated that EMT plays a key role in the process of tumor cell dissemination [17–20]. In consequence, tumor cells undergoing EMT may migrate into peripheral blood as CTCs. Due to their mesenchymal stemness features, these cells might be able to reach distant sites of the body and initiate metastases. In the following review we will discuss current data on the EMT and stem cell markers in CTCs of breast cancer and their clinical relevance.

## 2. Tumor Cell Dissemination and Its Role in the Metastatic Cascade

Distant metastasis represents the major cause of morbidity and mortality in breast cancer patients [21, 22]. Tumor cell dissemination is a phenomenon that occurs in the very early stage of carcinogenesis and is thought to be a potential source of metastatic disease [23]. Disseminated tumor cells in bone marrow can be detected in up to 30–40% of primary breast cancer patients at the time of diagnosis and are strongly associated with impaired prognosis [1]. Depending on the sensitivity of the assay used and stage of disease, the detection rates of CTCs in peripheral blood range from 10 up to 80%; prognostic relevance of CTCs has been recently confirmed by several clinical trials both in the adjuvant and in the metastatic setting. However, data on CTC prevalence and their clinical significance, especially in early breast cancer, are to date incoherent [24–37]. Hematogenous spread of tumor cells into blood circulation of patients with solid malignancies has been a known phenomenon for a long time [35, 38, 39]. While numerous tumor cells daily reach peripheral blood, only a small fraction of these cells has the ability to survive and to arrive at secondary homing sites “metastatic inefficiency” [11, 12]. Moreover, their seeding at the secondary sites is not a random process. As suggested by Paget in the “seed and soil” hypothesis from 1889 and confirmed by several studies, the interactions between circulating tumor cells “seeds” and the microenvironment of their potential homing sites “soil” play a crucial role in the formation of metastasis [38, 40–42]. These findings are in accord with clinical data; a pooled analysis of nine studies involving 4703 primary breast cancer patients demonstrated that more than half of patients with disseminated tumor cells in bone marrow at the time of diagnosis do not develop metastatic disease [1]. CTCs seem to represent a highly heterogeneous cell population with regard to their morphology, molecular characteristics, implantation efficiency after dissemination and their metastatic potential [43–45].

## 3. EMT/MET

Epithelial-mesenchymal transition is a process well known from embryogenesis. In order to reach their final destination, embryonic epithelial cells acquire functional and phenotypic properties of migratory, invasive mesenchymal cells and thus become detached from the surface of the embryo [46, 47]. Interestingly, epithelial-mesenchymal transition represents a reversible mechanism; once the target localization has been reached, these cells undergo a reverse process of mesenchymal epithelial transition (MET) and recover their epithelial character to proliferate and form differentiated tissues [48]. This phenomenon, essential for embryonic development, has been recognized to represent a crucial step in tumor progression and metastasis [13].

The process of EMT involves the loss of cell-to-cell adhesions, loss of apicobasal cell polarity, and increment of migratory and invasive features of mesenchymal cells [48]. EMT can therefore compromise the mechanical integrity of the tissue [49]. EMT, once induced in tumor cells, may allow them to escape from primary tumor, migrate through the blood unaffected by therapeutic agents, and reach the site of future metastasis. Furthermore, it has been postulated that MET also represents the part of metastatic formation and that tumor cells regain their epithelial properties at their secondary homing sites [50, 51]. This hypothesis is in accord with the observation that metastatic lesions generally share epithelial features of the primary tumor (e.g., E-cadherin expression) [52, 53].

EMT process can be induced by extracellular factors like transforming growth factor *β* (TGF*β*), Wnt, Notch, epidermal growth factor (EGF), hypoxia, and others [48]. Numerous transcription factors inducing EMT, like SNAIL, TWIST, SLUG, ZEB1, ZEB2, and FoxC2, have been evaluated [54]. Loss of E-cadherin, overexpression of N-cadherin, and cytoskeletal alterations (e.g., expression of vimentin) hallmark this process causing phenotypical and structural changes that lead to acquisition of motility and invasiveness of cells that have undergone EMT. Several studies have shown a correlation between EMT process and high aggressiveness of breast cancer. EMT markers seem to be associated with basal-like breast cancer phenotype and, therefore, with high invasiveness and metastatic potential [55, 56].  Table 1 summarizes markers used for detection and characterization of CTCs showing epithelial as well as mesenchymal phenotypes.

## 4. Detection of Tumor Cell Dissemination

The challenge in identifying and detecting CTCs is based on their rare number as well as the lack of a universal breast cancer marker. The majority of methods currently used are based on the detection of epithelial markers. The main disadvantage lies in the fact that cells undergoing EMT or with a mesenchymal phenotype might thus be missed. Only a few markers useful in the isolation of CTCs with a mesenchymal phenotype have been evaluated (Table 1). In the past ten years the number of assays to detect and characterize CTCs has increased steadily. All techniques have in common the fact that, due to the low frequency of the isolated tumor cells, they have to be extremely sensitive. In several cases the first step is the enrichment of tumor cells [57]. The choice of enrichment and characterization steps depending on the markers analyzed (especially EpCAM) is crucial to allow as well as to limit the detection of cells undergoing EMT or not. A short perception of enrichment and detection methods in regard to EMT and stem cell markers, some of them commercially available, will be given in the following. These methods are summarized in Table 2.

One way to enrich disseminated tumor cells is density gradient centrifugation. Mononuclear cells are isolated using Ficoll and are subsequently spun on glass slides. Visualization of the tumor cells beside the leukocytes is effected by means of immunocytochemistry. Due to the lack of a general marker, tumor cells are characterized as epithelial cells which are positive, among others, for EpCAM or cytokeratins [58]. Theodoropoulos et al. could identify CTCs with a putative stem cell-like phenotype in the blood of metastatic breast cancer patients using either cytokeratin, CD44, and CD24 or cytokeratin, ALDH1, and CD24 after density gradient centrifugation [59].

Another way to enrich CTCs is to label the cells with specific antibodies which are conjugated with magnetic particles. There are several tests commercially available which are based on the immunomagnetic enrichment of epithelial markers, especially EpCAM [24, 60], therefore limiting the possibilities to detect mesenchymal tumor cells which have undergone EMT. They differ in the subsequent characterization of the CTCs: commonly used techniques are the antibody-based detection of specific markers on the protein level and also on the RNA level using RT-PCR.

The semiautomatic CellSearch system (Janssen Diagnostics, Raritan, NJ, USA) which has been approved by the FDA is based on an immunomagnetic enrichment of epithelial cells using EpCAM-specific antibodies coated with magnetic beads. CTCs are quantified and further characterized by immunofluorescence detecting cytokeratins 8, 18, and 19 and CD45 to exclude leucocytes as well as staining of the nuclei (DAPI) [24, 61]. Additional staining of CD44 could be shown by Lowes et al. [62]. Using the CellSearch Profile Kit which consists only of the immunomagnetic enrichment step of EpCAM+ cells without further characterization allows the individual subsequent characterization of the CTC, using among others ALDH1 [63, 64].

Additional assays are commercially available to detect CTCs based on the analysis of the expression levels of epithelial or tumor-specific genes, where applicable with a preceding enrichment step. In case of the AdnaTest Breast Cancer (AdnaGen GmbH, Langenhagen, Germany) this enrichment step is performed using magnetic beads which are coated with EpCAM- and MUC1-specific antibodies. Subsequent RT-PCR allows the quantitative analysis of the expression levels of MUC1, GA733-2, and HER2 [60, 65, 66]. The additional characterization of the CTCs is effected by means of detection of the EMT and stem cell markers TWIST, Akt2, PI3K, and ALDH1, respectively [17, 20].

There are several approaches to enrich CTCs using special chips combining microfluidics and immobilization of CTCs by binding of specific antibodies (e.g., CTC-chip, Herringbone Chip) [67, 68]. The latter chip was used by Yu et al. to establish an RNA in situ hybridization assay to detect and quantify CTCs with either an epithelial or mesenchymal phenotype or with a phenotype in between (partial EMT). The expression levels of seven pooled epithelial transcripts (EpCAM; cytokeratins 5, 7, 8, 18, and 19 and cadherin 1) and three pooled mesenchymal transcripts (SERPINE1/PAI1, cadherin 2, and fibronectin 1) were analyzed to characterize CTCs which were detected by binding at least one of the following antibodies on a herringbone chip: EpCAM, HER2, or EGFR [69].

Another technique to enrich CTC which is solely based on the size of the cells is filtration. Several systems are available, for example, the ISET filter using pores with a diameter of 8 *μ*m [70]. The same pore size was used in another study combining Whatman Nuclepore track-etched membranes and immunofluorescent staining of cytokeratins 8, 18, and 19 as well as CD45 to exclude leucocytes [71].

Flow cytometry is another technique which allows an individual characterization of rare cells like CTCs. Using flow cytometry, Giordano et al. could detect a subpopulation of cancer stem cells expressing either ALDH1, CD44, and low amounts of CD24 or ALDH1 and CD133 [18].

Although the majority of assays use EpCAM as detection marker, different markers are currently used to detect and enrich CTC (Table 2). Due to the fact that CTCs change their phenotype during EMT and MET, false negative results can be obtained depending on which detection marker was used. EpCAM-based assays involve the risk that CTC showing a mesenchymal phenotype might be missed.

## 5. Can EMT Be Detected in CTCs?

To date, several methods have been developed to detect isolated tumor cells in peripheral blood and bone marrow of breast cancer patients. Since there is no breast cancer specific marker to identify these cells, most detection assays rely on their epithelial characteristics [72, 73]. Based on the assumption that the acquisition of a mesenchymal phenotype by a small fraction of tumor cells disseminated from primary tumor represents a crucial step in the metastatic cascade allowing these cells to migrate to their secondary homing sites and build metastasis, it is possible that EMT markers can be detected among the CTCs of breast cancer patients [13]. This hypothesis has been recently confirmed by various studies in both metastatic and early breast cancer [18–20, 20, 74–78]. Mego et al. demonstrated that EMT markers positive CTCs can be detected in up to 26% of metastatic breast cancer patients. Moreover, a high expression of EMT markers predicted shorter progression free survival in these patients [77]. Aktas et al. showed in their trial on 39 metastatic breast cancer patients that EMT markers, such as TWIST1, Akt2, and PI3K*α*, can be codetected in up to 62% of CTC positive blood samples; EMT markers were more likely to be found in patients resistant to therapy, suggesting increased invasiveness of tumor cells undergoing this process. Interestingly, cells undergoing EMT have also been detected in the blood of 7% of patients negative for CTCs [20]. Similar findings in primary breast cancer were presented by Kasimir-Bauer et al.; EMT markers could be detected in 72% of CTC positive and 18% of CTC negative patients, respectively [17]. Raimondi et al. demonstrated the expression of EMT markers (e.g., vimentin, fibronectin) in up to 38% of breast cancer patients tested by the standard definition as CTC negative [78]. These findings suggest that, in addition to CTCs expressing epithelial antigens, a fraction of CTCs with exclusively mesenchymal phenotype could exist and thus remain undetectable for assays based on epithelial character of these cells. However, due to the methodology, morphological features of the cells were not evaluated in these trials and false positive results cannot be excluded [17, 20]. In this regard, CTCs coexpressing mesenchymal and epithelial markers have been visualized in three other studies in breast cancer patients confirming that both kinds of markers can be expressed in the same cell [69, 75, 76]. Additionally, in the analysis by Armstrong et al. vimentin-positive CTCs were detected in peripheral blood of metastatic breast cancer patients while paired metastases from the same patients were shown to be negative for this marker [75]. This suggests a reversibility of the EMT process once tumor cells reach their destination resembling the phenomenon of epithelial plasticity known from embryonic development [48]. Available literature on EMT in CTCs of breast cancer patients is summarized in Table 3.

## 6. Are CTCs Cancer Stem Cells?

One recently discussed hypothesis indicates that tumor progression and metastatic spread can be traced to a small fraction of tumor cells with stem cell-like characteristics [81, 82]. These cancer stem cells have been identified in breast cancer tissue and were shown to be associated with tumors of aggressive behavior [83]. Assuming that CSCs are responsible for tumor cell dissemination and further metastasis, it seems likely that putative stem cell-like features should be found among tumor cells disseminated from primary tumor. This hypothesis has been confirmed by several researchers [17–20, 77–79]. As reported by Balic et al., most disseminated tumor cells in bone marrow of breast cancer patients presented with CD44^+^/CD24^−/low^ phenotype [79]. Moreover, it has been shown that DTCs with CD44^+^/CD24^−/low^ phenotype are associated with increased prevalence of metastases and with tumors characterized by aggressive biology [80, 84].

According to recent data both stem cell and EMT markers are frequently coexpressed in CTCs of breast cancer patients [18, 77]. These findings support the theory that EMT generates a cell population with stem cell-like features, a phenomenon that has been confirmed by numerous experimental trials [15, 85]. CTCs presenting stem cell-like characteristics have been found in both primary and metastatic breast cancer. In a recent study by Kasimir-Bauer et al. on 502 primary breast cancer patients 46% of CTC positive and 5% of CTC negative blood samples were positive for ALDH1, a common stem cell marker [17]. Similar findings have been shown by Aktas et al. in the metastatic situation. Moreover, a presence of stem cell-like CTCs in peripheral blood of breast cancer patients was shown to be associated with therapy resistance; stem cell markers or EMT factors or both were detected in 74% (25/34) of nonresponders and in 10% (2/21) of patients who responded to systemic treatment [20]. In the trial by Raimondi et al. an overexpression of stem cell markers in CTCs was correlated with advanced stage of disease [78]. Cancer stem cells are currently believed to be the cause of therapy resistance and treatment failure in breast cancer [86]. Data on stem cells markers in CTC of breast cancer patients are summarized in Table 4.

## 7. Therapeutic Consequences

To date, systemic therapies target either highly proliferative tumor cells (cytotoxic therapy) or cells with a specific phenotype (e.g., HER2-targeted treatment). However, such therapies are not able to identify cells that act as a source for subsequent metastasis in a selective manner. Tumor cells with putative stem cell-like expression profile are assumed to enter the blood circulation early in the course of disease and might elude therapy precisely because of their stem cell character [20, 75]. Hypothetically, specific elimination of these cells could prevent the colonization of secondary homing sites and metastasis formation. Thus, the potential existence of a stem cell-like cancer cell might lead to a paradigm shift in oncologic treatment.

Detection and characterization of CTCs have become an important focus of oncologic research; several clinical trials have been initiated during the last decade that evaluate not only CTCs within accessory translational projects, but also ones that focus exclusively on CTCs and stratify therapy according to CTC levels [87]. Most of these trials (e.g., SWOG0500, CirCe01, TREAT CTC, and DETECT III and IV) are based on immunocytochemical detection of CTCs using the FDA-approved CellSearch system (Veridex, Warren, NJ, USA), a semiautomated antibody-based quantitative technique [88]. Since CTCs are enriched by immunomagnetic beads linked with anti-EpCAM antibodies and detected using antibodies against epithelial antigens, loss of epithelial markers during EMT could make these cells “invisible” to the assay and possibly influence treatment decisions [78, 89]. Gorges et al. reported that use of EpCAM-based enrichment techniques may lead to failure in CTC detection; in an animal based model EpCAM-based AdnaTest failed to detect CTCs despite clinically apparent metastasis. However, CTCs could be detected by PCR without the enrichment step [89]. Recently, antibody-based therapies against tumor cells expressing epithelial markers have been introduced in the treatment of cancer of epithelial origin. Catumaxomab, a trifunctional antibody directed against EpCAM, is a potent therapeutic agent for malignant ascites in EpCAM-positive advanced cancer (e.g., ovarian cancer) [90, 91]. Since EMT involves at least temporary downregulation of EpCAM expression, it might influence the efficacy of EpCAM-directed therapy on tumor cells undergoing EMT.

Therefore, signaling pathways involved in EMT and responsible for the formation of CSCs represent potential targets for future treatment regimens, and drugs inhibiting these pathways are being tested in preclinical and clinical trials [92]. In this regard everolimus (RAD001), an oral inhibitor of PI3K/Akt/mTOR pathway, was shown to inhibit cancer stem cells in vitro and in vivo and demonstrated potential effectivity in treatment of breast cancer cells resistant to standard therapy possibly through this mechanism[93–95]. These data are in accordance with clinical results; in a phase II study RAD001 was shown to restore sensitivity to tamoxifen in metastatic breast cancer patients with endocrine resistance improving the clinical benefit rate at six months in these patients [96]. A phase III BOLERO-2 trial demonstrated a 6-month improvement in progression-free survival in patients with resistance to nonsteroidal aromatase inhibitor treated with everolimus in combination with exemestane versus exemestane alone [97]. Everolimus is currently being evaluated for its potential to overcome trastuzumab resistance as well. A phase III BOLERO-1 trial compares trastuzumab and paclitaxel with and without everolimus, while the phase III BOLERO-3 trial compares trastuzumab and vinorelbine with and without everolimus.

Hedgehog, Notch, and Wnt represent further signaling pathways involved in formation of breast cancer stem cells [98–100]. Since the expression of Notch ligands has been demonstrated to be significantly elevated in triple negative breast cancer, Notch has become a promising target in breast cancer treatment [101]. In this context blocking of Notch by *γ*-secretase inhibitors (GSIs) has been the most extensively used approach. GSIs were shown to induce apoptosis and decrease proliferation in breast cancer cell lines and to eliminate breast cancer stem cells in vitro [102, 103]. GSIs like MK-0752 or RO4929097 have been tested in phase I and II clinical trials in primary and metastatic breast cancer providing early clinical evidence of effectiveness for these agents in breast cancer therapy [104, 105]. A phase I study analyzes RO4929097 in combination with Hedgehog pathway antagonist vismodegib in metastatic breast cancer patients [106]. Vismodegib, established in the therapy of advanced basal cell carcinoma, was also shown to inhibit tumor cell growth in tamoxifen resistant breast cancer in vivo and in vitro [107]. Furthermore, PKF118-310 an inhibitor of Wnt signaling pathway was recently reported to eradicate breast cancer stem cells in a mouse model overexpressing HER2, thus also representing a potential drug candidate for the treatment of breast cancer [108].

An additional agent that was demonstrated to be effective against breast cancer stem cells is all transretinoic acid (ATRA). In a recent experimental approach, ATRA was able to eliminate breast cancer cells that gained CSC properties, suggesting its effectiveness in cancer resistant to conventional oncologic therapies [109]. However, ATRA has to date performed poorly in clinical trials; in a pilot phase II study 17 metastatic or recurrent breast cancer patients were treated with ATRA in combination with paclitaxel showing time to progression and survival rates similar to those reported for paclitaxel alone [110].

Another promising drug candidate in this context is salinomycin, which was shown to inhibit tumor growth in mice by eradicating breast cancer stem cells [111]. Recent preclinical trials demonstrated that salinomycin is particularly effective against cancer growth in combination with conventional chemotherapeutics, supporting the postulation that targeting different cell populations is essential in cancer therapy [112].

## 8. Conclusions

Multiple studies have shown that single tumor cells undergo transdifferentiation which enables intravasation; this important step of metastatic cascade is termed epithelial-mesenchymal transition. Through EMT, circulating tumor cells downregulate epithelial antigens and cell-to-cell adhesion and thus enhance their motility and invasive potential. Cells that undergo EMT seem to gain stem cell-like properties; such cells represent a small fraction of tumor cells capable of self-renewal and highly resistant to cytotoxic treatment. Since the majority of CTC detection systems are based on the presence of epithelial markers, tumor cells that have undergone EMT might elude classical detection methods, which may lead to false-negative results.

## Figures and Tables

**Table 1:** CTC detection and characterization markers.

Marker	Reference	CTC detection or enrichment marker	Epithelial marker	Mesenchymal marker	Stem cell marker
Akt2	[17, 19, 20]			x	
ALDH1	[17–20, 59, 64, 77, 78]				x
Bmi1	[19]				x
CD133	[18]				x
CD24	[18, 59, 77]				x
CD44	[18, 19, 59, 62, 77]				x
Cytokeratins 8, 18, 19	[24, 61]	x	x		
E-cadherin (Cadherin 1)	[69]		x		
EGFR	[69]	x	x		
EpCAM (GA733-2)	[24, 58, 60, 61, 65, 69]	x	x		
Fibronectin 1	[69, 78]			x	
FoxC2	[54, 74]			x	
HER2	[60, 65, 69]	x	x		
MUC1	[60, 65]	x	x		
N-cadherin (Cadherin 2)	[69, 75]			x	
pan-Cytokeratin	[59, 69]		x		
PI3K	[17, 19, 20]			x	
SERPINE1/PAI1	[69]			x	
SLUG	[54, 74]			x	
SNAIL 1	[18, 54, 74, 77]			x	
TG2	[18]			x	
TWIST 1	[17–20, 54, 74, 76, 77]			x	
Vimentin	[75, 76, 78]			x	
ZEB1	[18, 54, 74]			x	
ZEB2	[54]			x	

**Table 2:** Detection and characterization methods of CTCs.

Method	Reference	Based on	Detection marker	Characterization marker
AdnaTest Breast Cancer	[60, 65, 66]	PCR	EpCAM, MUC1	MUC1, GA733-2, HER2
AdnaTest EMT-1/stem cell	[17, 20]	PCR	EpCAM, MUC1	TWIST, Akt2, PI3K, ALDH1
CellSearch CTC Kit	[24, 61, 62]	Antibody	EpCAM	CK 8, CK 18, CK 19, CD45, DAPI, HER2, EGFR, CD44
CellSearch Profile Kit	[63, 64]	Antibody	EpCAM	To be determined; for example, ALDH1
CTC-Chip	[67]	Antibody	EpCAM	Cytokeratin, CD45, DAPI
Ficoll/immunocytochemistry	[58, 59, 73, 79]	Antibody	To be determined; for example, EpCAM, Cytokeratin	To be determined; for example, CD44, CD24, ALDH1
Filtration	[70, 71]	Filtration	To be determined; for example, CK 8, CK 18, CK 19, CD45	To be determined
Flow cytometry	[80]	Antibody	EpCAM, ALDH1	CD44, CD24
Herringbone-chip	[68, 69]	Antibody	EpCAM, HER2, EGFR	EpCAM, CK 5, CK 7, CK 8, CK 18, CK 19, cadherin 1, cadherin 2, SERPINE1/PAI1, fibronectin 1

**Table 3:** EMT markers in CTC of breast cancer patients.

Author	Year	*N*	Method	EMT marker	Expression rate in CTC
Kasimir-Bauer et al. [17]	2012	502^1^	RT-PCR	TWIST1, Akt2 PI3K*α*	72%^3,∗^, 18%^4,∗^
Giordano et al. [18]	2012	28^2^	RT-PCR	TWIST1 SNAIL1 ZEB1 TG2	88%*
Barriere et al. [19]	2012	24^1^	RT-PCR	TWIST1 Akt2 PI3K*α*	13% 13% 67%
Mego et al. [77]	2012	21^2^	RT-PCR	TWIST1 SNAIL1	26% 21%
Armstrong et al. [75]	2011	16^2^	IFC	Vimentin N-cadherin	70% 67%
Kallergi et al. [76]	2011	50^1,2^	IFC	TWIST1 Vimentin	73%^1^, 100%^2^ 77%^1^, 100%^2^
Mego et al. [74]	2011	52^1^	RT-PCR	TWIST1 SNAIL1 SLUG ZEB1 FoxC2	15,4%*
Raimondi et al. [78]	2011	92^1,2^	RT-PCR	Vimentin Fibronectin	28%^3^, 38%^4^ 18%^3^, 35%^4^
Aktas et al. [20]	2009	39^2^	RT-PCR	TWIST1 Akt2 PI3K*α*	62%^3,∗^, 7%^4,∗^

^1^Primary breast cancer, ^2^metastatic breast cancer,^ 3^CTC positive group, ^4^CTC negative group; *at least one EMT marker was expressed, PFS: progression free survival.

**Table 4:** Stem cell markers in CTC of breast cancer patients.

Author	Year	*N*	Method	Stem cell marker	Expression rate in CTC
Kasimir-Bauer et al. [17]	2012	502^1^	RT-PCR	ALDH1	46%^3^, 5%^4^
Giordano et al. [18]	2012	28^2^	Flow cytometry	ALDH CD44^+^/CD24^low^	0.1% 3%^3,∗^, 49%^4,∗^
Barriere et al. [19]	2012	24^1^	RT-PCR	ALDH1 CD44 Bmi1	54% 67%** 33%**
Mego et al. [77]	2012	17^2^	Flow cytometry	ALDH CD44^+^/CD24^low^	n. d.
Raimondi et al. [78]	2011	61^1,2^	RT-PCR	ALDH1	46%
Aktas et al. [20]	2009	39^2^	RT-PCR	ALDH1	69%^3,∗^, 14%^4,∗^
Theodoropoulos et al. [59]	2010	30^2^	IFC	ALDH1 CD44^+^/CD24^low^	18% 35%

^1^Primary breast cancer, ^2^metastatic breast cancer,^ 3^CTC positive group, ^4^CTC negative group; *among ALDH positive cells, **among EMT or ALDH1 positive cells, n. d.: not done, IFC: immunofluorescence.

## Abbreviations

ALDH: Aldehyde dehydrogenase
CSC: Cancer stem cell
CTC: Circulating tumor cell
DAPI: 4′,6-Diamidino-2-phenylindole
DTC: Disseminated tumor cell
EGF: Epidermal growth factor
EGFR: Epidermal growth factor receptor
EMT: Epithelial-mesenchymal transition
EpCAM: Epithelial cell adhesion molecule
GA733-2: Gastrointestinal tumor-associated antigen
HER2: Human epidermal growth receptor 2
MET: Mesenchymal epithelial transition
mTOR: Mammalian target of rapamycin
MUC1: Mucin 1
PCR: Polymerase chain reaction
PI3K: Phosphoinositide 3-kinase
TGF*β*: Transforming growth factor beta.
